# Characterization of an Insoluble and Soluble Form of Melanin Produced by *Streptomyces cavourensis* SV 21, a Sea Cucumber Associated Bacterium

**DOI:** 10.3390/md20010054

**Published:** 2022-01-06

**Authors:** Joko Tri Wibowo, Matthias Y. Kellermann, Lars-Erik Petersen, Yustian R. Alfiansah, Colleen Lattyak, Peter J. Schupp

**Affiliations:** 1 Institute for Chemistry and Biology of the Marine Environment (ICBM), Carl von Ossietzky University Oldenburg, Schleusenstr. 1, 26382 Wilhelmshaven, Germany; lars-erik.petersen@weizmann.ac.il; 2 Research Center for Biotechnology, National Research and Innovation Agency (BRIN), Jl. Raya Bogor KM 46, Cibinong 16911, Indonesia; 3 Research Center for Oceanography, National Research and Innovation Agency (BRIN), Jl. Pasir Putih 1, Ancol Timur, Jakarta Utara 14430, Indonesia; yustian.alfiansah@awi.de; 4 Center for Aquaculture Research (ZAF), Alfred Wegener Institute (AWI), Helmholtz Center for Polar and Marine Research, Am Handelshafen 12, 27570 Bremerhaven, Germany; 5 DLR Institute of Networked Energy Systems, 26129 Oldenburg, Germany; Colleen.Lattyak@dlr.de; 6 Helmholtz Institute for Functional Marine Biodiversity at the University of Oldenburg (HIFMB), Ammerländer Heerstrasse 231, 26129 Oldenburg, Germany

**Keywords:** melanin pigment, *Streptomyces*, water soluble, quorum sensing inhibition, antioxidant

## Abstract

Melanin is a widely distributed and striking dark-colored pigment produced by countless living organisms. Although a wide range of bioactivities have been recognized, there are still major constraints in using melanin for biotechnological applications such as its fragmentary known chemical structure and its insolubility in inorganic and organic solvents. In this study, a bacterial culture of *Streptomyces cavourensis* SV 21 produced two distinct forms of melanin: (1) a particulate, insoluble form as well as (2) a rarely observed water-soluble form. The here presented novel, acid-free purification protocol of purified particulate melanin (PPM) and purified dissolved melanin (PDM) represents the basis for an in-depth comparison of their physicochemical and biological properties, which were compared to the traditional acid-based precipitation of melanin (AM) and to a synthetic melanin standard (SM). Our data show that the differences in solubility between PDM and PPM in aqueous solutions may be a result of different adjoining cation species, since the soluble PDM polymer is largely composed of Mg^2+^ ions and the insoluble PPM is dominated by Ca^2+^ ions. Furthermore, AM shared most properties with SM, which is likely attributed to a similar, acid-based production protocol. The here presented gentler approach of purifying melanin facilitates a new perspective of an intact form of soluble and insoluble melanin that is less chemical altered and thus closer to its original biological form.

## 1. Introduction

Melanins are dark pigments with diverse structure that derived by the oxidation and polymerization of tyrosine in animals or phenolic compounds in lower organisms [1]. Melanins are classified into four groups: (1) eumelanins, (2) pheomelanins, (3) neuromelanins, and (4) pyomelanins. Eumelanins are black to brown insoluble melanin pigments that derived at least in part from the polymerization of L-dopa via 5,6-dihydroxindole intermediates (*cf.* Figure S1). Phaeomelanins have a yellow to reddish brown color, are alkali soluble, contain sulfur, and are derived from the oxidation of cysteinyldopa precursors via benzothiazine and benzothiazole intermediates. Neuromelanins are dark pigments produced within neurons by the oxidation of dopamine and other cathecolamine precursors. Pyomelanins are dark pigments produced by microorganisms mainly through homogentisate. In many bacteria, including *Streptomyces* spp., melanin serves as a natural marker for taxonomic identification up to the genus level [1,2,3]. Specifically, the production of melanin in *Streptomyces* was assigned to an adaptive response to elevated environmental stress conditions such as light, temperature, oxidative stress, as well as an elevated concentration of heavy metals [4,5,6]. In addition, melanin produced by *Streptomyces* spp. possesses various bioactivities (i.e., antibiotic, anticancer and antioxidant activities) [7,8,9,10].

Most commonly, melanin is either derived from animals such as the ink from cuttlefish or chemically synthesized from the oxidation of tyrosine with hydrogen peroxide [11]. Thus, the bacterial production of melanin by *Streptomyces* spp. may be a promising alternative to produce sustainable, somewhat inexpensive, and industrial feasible levels of melanin. Two previous studies optimized the melanin production in *Streptomyces* bacteria. *Streptomyces glaucescens* NEAE-H, an Actinobacterium isolated from Egyptian soil, produced 316.5 mg L^−1^ of extracellular melanin under optimized media and culture conditions [7]. *Streptomyces kathriae* produced 13.7 g L^−1^ melanin under optimal medium and culture conditions [12]. However, the latter studies used boiling acid to precipitate the melanin. Even though the acid precipitated melanin shares similar physicochemical characteristics with synthetic melanin, the use of acid and high temperatures is considered a harsh and destructive treatment [11]. Indeed, the acid precipitated treatment significantly altered and partly decomposed the molecular structure of the melanin polymer by extensive decarboxylation reactions [13]. Furthermore, melanin derived by organic synthesis or acid precipitation of bacterial melanin resulted in forms of melanin that neither dissolved in common organic- and/or inorganic solvents. Nevertheless, a soluble form of melanin is highly desirable for potential commercial biotechnological applications [14,15].

In this study, we report the production and different purification pathways of both the insoluble and soluble form of melanin found in *Streptomyces cavourensis* SV 21 and, moreover, characterize and compare both types of melanin using a variety of physiochemical and bioactivity assays. In a previous study [16], crude extracts of this strain showed strong bioactivity against the human hepatitis C virus (HCV) and the multiple drug resistance (MDR) microorganisms *Staphylococcus aureus*, *Bacillus subtilis*, and *Mucor hiemalis*. A prior isolation and structure elucidation of the bioactive antibiotic valinomycin and three derivatives produced by the same strain SV 21 had been previously described in 2021 by Wibowo and colleagues [17]. This study focusses exclusively on the characterization and bioactivities of the insoluble and soluble melanin pigments derived from *S. cavourensis* SV 21.

## 2. Results and Discussion

### 2.1. Growth Characteristics of the Bacterial Culture S. cavourensis SV 21 within the Liquid Marine Broth (MB) and on Marine Agar (MA)

**Figure 1:**
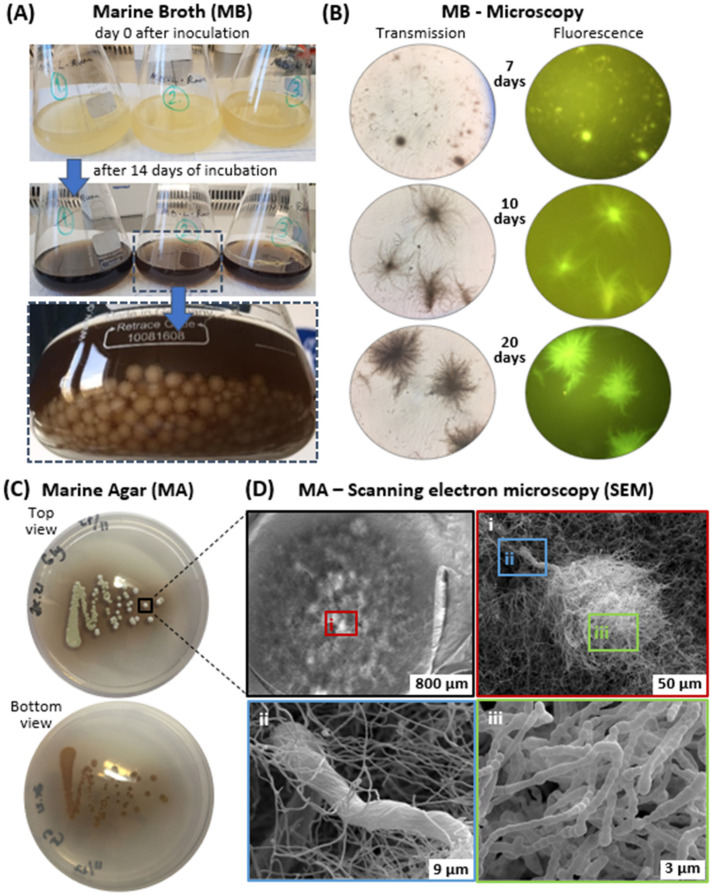
(**A**) Color of three replicates of *Streptomyces cavourensis* SV 21 cultures grown in marine broth (MB) at day 0 and after 14 days of incubation. (**B**) Transmission and fluorescence light microscopy (magnification 10 × 100, oil immersion) pictures of *S.* *cavourensis* SV 21 colonies. Here, colonies were grown in liquid MB media shaken at 22 °C and sampled at 7, 10, and 20 days. (**C**) Top and bottom view of 5-day old *S. cavourensis* SV 21 cultures on marine agar (MA). (**D**) SEM pictures of a 5-day old colony of *S. cavourensis* SV 21 grown on MA media and cultured at a temperature of 22 °C (single bacterial colony shown); (**i**) to (**iii**) represent further magnifications into the bacterial culture down to the cellular, µm-level. In (**i**) substrate and aerial mycelia are shown, (**ii**) zooms into the substrate mycelium and the main stem of the aerial mycelia, and (**iii**) magnifies on the spore-chains of *S. cavourensis* SV 21.

*S. cavourensis* SV 21 grew on marine agar (MA) as well as in marine broth (MB) media (*cf.* Figure 1) and quickly developed its characteristic earthy smell, which derived from the organic molecule geosmin [18]. During incubation, the bacterium secreted a dark-brown pigment into both MA and MB. However, we were unable to separate the dark-brown pigments from the liquid media, neither by intense centrifugation (i.e., 10,000 rpm for 30 min) nor through extraction with water-immiscible organic solvents (i.e., ranging from non-polar solvents like *n*-hexane to dichloromethane to polar solvents such as *n*-butanol). Additionally, the water-soluble pigments could pass through a 0.45 µm filter, but not through a 0.2 µm filter. Hence, it can be classified as dissolved organic matter (DOM) [19].

Already in 1972, Arai and Mikami termed these dark-brown water-diffusible pigments as melanoid or melanin pigments [2]. Other members of the *Streptomyces* genus (i.e., *S. albolongus*, *S. bacillaris*, *S. cyaneofuscatus*, *S. fimicarus*, *S. grisseobrunneus*, *S. griseus* subs. *Griseus, S. lavendulae* subs. *Lavendulae*, and *S. luridiscabiei*) also showed production of these water-soluble, melanoid pigments [20].

In MB media, bacterial colonies possessed spherical forms that became bigger and more filamentous as they grew (Figure 1A). Cells of *S. cavourensis* SV 21 could be visualized by transmission light microscopy as well as fluorescence microscopy. By using a blue laser (488 nm) the bacterial colonies emitted an intense greenish fluorescence (Figure 1B). Interestingly, the melanin pigmentation did not affect the whitish appearance of the outer layer of the colony in both MB and MA media. It may be possible that bacterial cells had actively produced oxidative agents or enzymes that oxidized melanin on the cell’s surface [21]. The dark-brown coloration was also noticeable on the agar plates inoculated with *S. cavourensis* SV 21. The intensity of the pigmentation was highest in close vicinity to the colony and was fading with greater distance from the colony (*cf.* Figure 1C). Within the bacterial colony itself, a well-developed substrate and aerial mycelium was formed. The color of mature aerial mycelia was also greyish white with some clear liquid drops on top of the colonies. The backside of the petri dish showed substrate mycelia with a yellowish-white color. Observation of a colony of the bacterium under SEM exhibited Rectiflexibiles-type (straight) and flexuous spore chains (*cf.* Figure 1D).

### 2.2. Physicochemical Characteristics of S. cavourensis SV 21 Supernatant

During the 14-day long incubation experiment, the culture constantly expelled dark-brown pigment to the aqueous environment at all tested conditions (i.e., at 22 °C, 30 °C, under constant light, as well as complete darkness; Figure S2). The pigment was accumulating with the onset of the exponential bacterial growth phase. The production of the pigment in MB medium was faster at higher temperatures. This is in line with a previous study, showing that the pigment production in *Streptomyces cavourensis* strain RD8 was highest at 30 °C, thereby representing the optimum growth temperature for this particular strain [10].

Intriguingly, the accumulation of the pigment occurs simultaneously with an increase in pH (Figure S3). Although it is not clear whether the constant pigment production is responsible for the constantly rising pH values, melanin is known for its vast numbers of negative surface charges [22].

**Figure 2:**
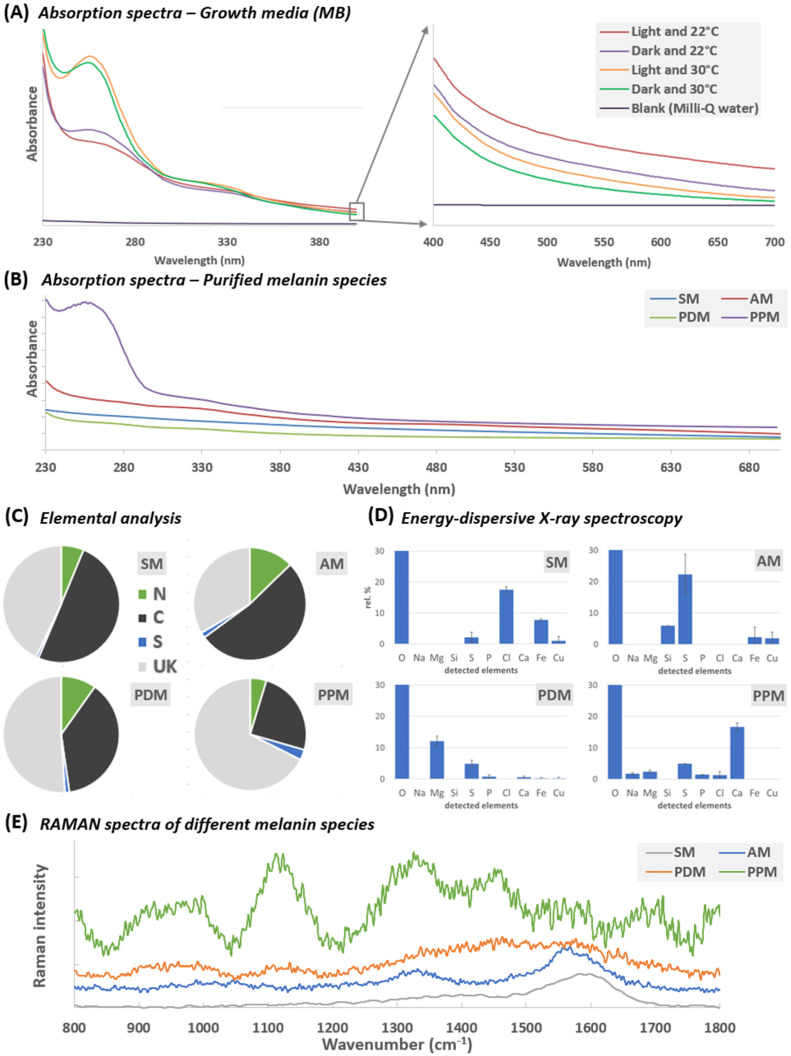
Physicochemical characteristics of the MB growth media (**A**) and the purified acid-based melanin (AM), purified particulate melanin (PPM), purified dissolved melanin (PDM) and a commercially available, synthetic melanin standard (SM; **B**–**E**). (**A**) shows the absorption spectra in the UV (230–400 nm) and visible light range (400–700 nm) of a Milli-Q blank (black line, negative control) and the supernatants of four different 13-day old incubations of *S. cavourensis* SV 21 (i.e., with and w/o light at 22 and 30 °C). In (**B**) the absorbance spectra (230–700 nm) of AM, PPM, and PDM purified from *S. cavourensis* SV 21 (*cf.* Figure 3) were compared to SM. For that, all samples were treated with 1N NH_4_OH to rise the pH to 12 and thus increasing the solubility properties of the otherwise poorly or insoluble melanin samples PPM, AM, and SM (*cf.* Table 1). The elemental composition of the different melanin types SM, AM, PDM, and PPM were determined using both, an elemental combustion (**C**) and energy dispersive X-ray (**D**) analyzer. In (**E**) the RAMAN spectra of the dried melanin powder AM, PDM, and PPM were compared to SM. For all samples the laser was set to 488 nm.

At the end of the incubation period of *S. cavourensis* SV 21, we analyzed the absorption spectra of the supernatant. The spectra in Figure 2A show that light was largely absorbed in the UV-C (230–290 nm), UV-B (290–320 nm), and UV-A (320–400 nm) range. However, if compared against a negative control (Milli-Q) the dark-colored supernatant also retained low absorption covering the entire visible range (400–700 nm; Figure 2A). Furthermore, in all experiments (i.e., 22 °C and 30 °C with and without light) two characteristic absorption peaks at ca. 255 nm and 330 nm were noticeable. Overall, the light absorption as an indicator of pigment production was higher in the supernatant from 30 °C compared to the 22 °C experiment, also indicating an optimum growth temperature at 30 °C for this particular strain [10].

The absorption spectra of the supernatants, showing a particular high absorbance of light in the UV-B and UV-A region, matches with reported literature data on melanin [23]. However, depending on the molecular composition of the complex structure of melanin, the absorption spectra may vary. In previous studies, the highest recorded absorption peak of melanin ranged between 250–400 nm [7,10,22] and thus matches our data.

In order to track the pigment production, we selected a wavelength of 340 nm as used in a previous study by [22]. The wavelength of 340 nm minimizes the number of possible interference with unwanted components such as nucleic acids, lipids, and/or proteins [22]. In addition, the wavelength is close to the range of the main building blocks of melanin (i.e., 5,6-dihydroxyindole-2-carboxylic acid, Figure S1) that holds an absorption peak at 321 nm [24]. Observation of the pigment production in each group showed an increase in absorbance at 340 nm with ongoing incubation (Figure S2). In day 1–5, the pigment production was faster at 30 °C than at 22 °C (Figure S2A,B). Light exposure seems to have a negative effect on the production of melanin at both, 22 °C and 30 °C (Figure S3). However, the magnitude of light absorbance at 340 nm after 13 days showed no significant difference among all four treatments.

### 2.3. Purification of the Two Distinct Melanin Forms Produced by S. cavourensis SV 21: Water-soluble (WSM) and Particulate Melanin (PM)

**Figure 3:**
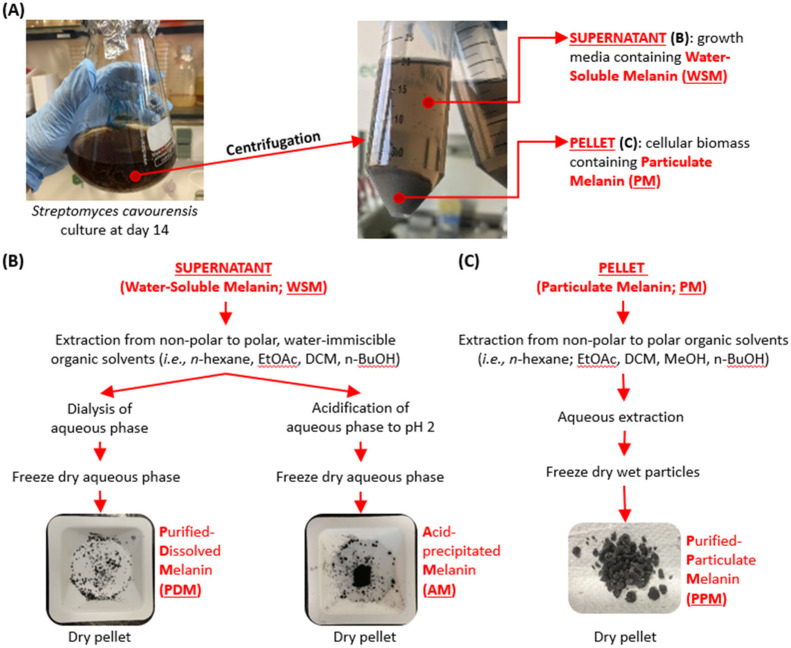
A schematic workflow illustrating how the different melanin types of *S. cavourensis* SV 21 were obtained. (**A**) shows the first physical separation step by centrifugation to achieve the water-soluble form of melanin (WSM) and the cellular/particulate form of melanin (PM). In (**B**) a step-by-step purification protocol is shown for WSM and in (**C**) for PM.

*S. cavourensis* SV 21 produced two distinct forms of melanin-like pigments: (1) a water-soluble form that neither could be extracted with polar or non-polar organic solvents, nor separated by intense centrifugation (10,000 rpm) nor through filtration through a 0.45 µm filter; and (2) a particulate form of melanin that accumulated together with the cell debris after centrifugation as a dark pellet. Like WSM, PM was neither extractable in any tested organic solvent nor in water. In the past, soluble bacterial melanin has often been precipitated out of solution using a low pH environment [6,7], whereas insoluble, particular melanin could partly be resolubilized in a strong basic solution [25]. However, the latter rather harsh chemical transformation reactions clearly influence the molecular structure of the melanin derivatives. Here, we present a more sensitive approach to gradually purify WSM into purified-dissolved melanin (PDM) and PM into purified-particulate melanin (PPM; cf. Figure 3) without substantially altering their chemical structure. For comparison, we also generated AM out of WSM and obtained a synthetic melanin (SM).

To further purify WSM and PM, the supernatant and the pellets were first washed with various organic solvents (Figure 3B,C, respectively). While for WSM only water immiscible solvents were used, PM was also extracted with methanol (MeOH). PM was further washed with Milli-Q and the remaining cell pellets lyophilized and stored at −20 °C for further analysis. The extracted material of WSM was separated into PDM and AM. For PDM we used a dialysis bag for removing dissolved contaminants and free salts having a molecular weight smaller than 3.5 K. The dark-brown pigment however, remained within the dialysis bag, while the unwanted dissolved compounds were flushed out through the semi-permeable membrane. AM was prepared as described in [6,7].

After the purification of WSM, the resulting dried PDM and AM were similar in shape and color. However, the amount of PDM (dialysis bag) was with 670 mg L^−1^ supernatant significantly higher than the 116 mg L^−1^ derived from supernatant from acid precipitation. In comparison, the amount of acid-precipitated pigment was lower than the amount of melanin from *Streptomyces glaucescens* NEAE-H (i.e., 317 mg L^−1^; [7]), and comparable to *S. cavourensis* RD 8 (i.e., 116 mg L^−1^; [10]). We want to highlight that PDM derived by dialysis has not been reported before and its yield is to this point unprecedented high. All three purified melanin fractions were kept in the dark at −20 °C to avoid photo-chemical degradation. Next, we compared the physiochemical characteristic of the purified forms of melanin (i.e., PDM, AM, and PPM) among each other and with a commercially available melanin standard (SM).

### 2.4. Characterisation and Comparison of SM, AM, PDM, and PPM

#### 2.4.1. Solubility Properties of SM, AM, PDM, and PPM in Organic and Inorganic Solutions

All tested melanin types (i.e., AM, PDM, PPM, and SM) were insoluble in all tested polar and non-polar organic solvents (*cf.* Table 1). On the other hand, PDM was easily soluble in all tested aqueous solutions, while SM and AM only showed good solubility in a strong alkaline solution (>pH 12), confirming previous reports (i.e., [15,23]). PPM was only partly soluble in the here tested aqueous solutions.

Most recently, a bacterial, water-soluble melanin became an emerging topic in the field of melanin research, since melanin has been described as practically insoluble in any organic and inorganic solvent [23]. Kiran and colleagues (2017) identified a nano-sized, water-soluble melanin (WSM) source from *Pseudomonas* sp. that showed a direct application as an antimicrobial agent [26]. Also, Silva and coworkers (2019) identified WSM from the Antarctic strain *Streptomyces fildesensis* that revealed great potential in solar-cell research [27].

Previous studies often used an acid-based treatment to obtain a water-soluble form of melanin. However, by treating a melanin containing sample with low pH, the vastly anionic polymer loses the ionic connection between itself and other adjoining organic compounds such as amino acids and/or polysaccharides. In this sense, Kimura and colleagues (2015) showed that after the acid precipitation process of a melanin sample derived from the Antarctic bacterium *Lysobacter oligotrophicus*, the remaining aqueous solution enriched in free polysaccharides such as n-acetyl-d glucosamine (65%, mol/mol), mannose (21%), and glucose (14%). They calculated that one molecule of melanin was approximately interacting with 114 molecules of polysaccharides [28]. Wold et al. (2020) treated the water-soluble melanin fraction, derived from the fungi *Inonotus obliquus,* with acid and analyzed the polysaccharide content. The fraction contained glucose, galactose, xylose, galacturonic acid, and mannose in a 5:2:2:2:1 ratio [29]. Another study by Aghajanyan and colleagues (2005) investigated the amino acid contend after hydrolyzation of the soluble melanin fraction of *Bacillus thuringiensis* and detected in total 20% amino acids from the total melanin weight, which were composed of aspartic acid, methionine, threonine, isoleucine, serine, leucine, glutamic acid, tyrosine, proline, phenylalanine, glycine, alanine, valine, arginine HCl, histidine HCl, and lysine HCl [14]. The latter studies demonstrated that the melanin composition is likely species specific and above all highly diverse in its molecular composition with infinite possibilities of combinations of amino acids and other organic molecules. Furthermore, it is important to note that a harsh chemical treatment, such as the often-used acid-hydrolysis, drastically changes the complex molecular composition and interaction among monomers of melanin, which in turn could have significant effects on the reactivity and bioactivity of the molecule. For comparison, in this study we developed a none-acid based, gentle purification method for bacteria derived, water-soluble melanin that is readily available and thus an inexpensive source for future biotechnological applications.

#### 2.4.2. Scanning Electron Microscopy (SEM)

**Figure 4:**
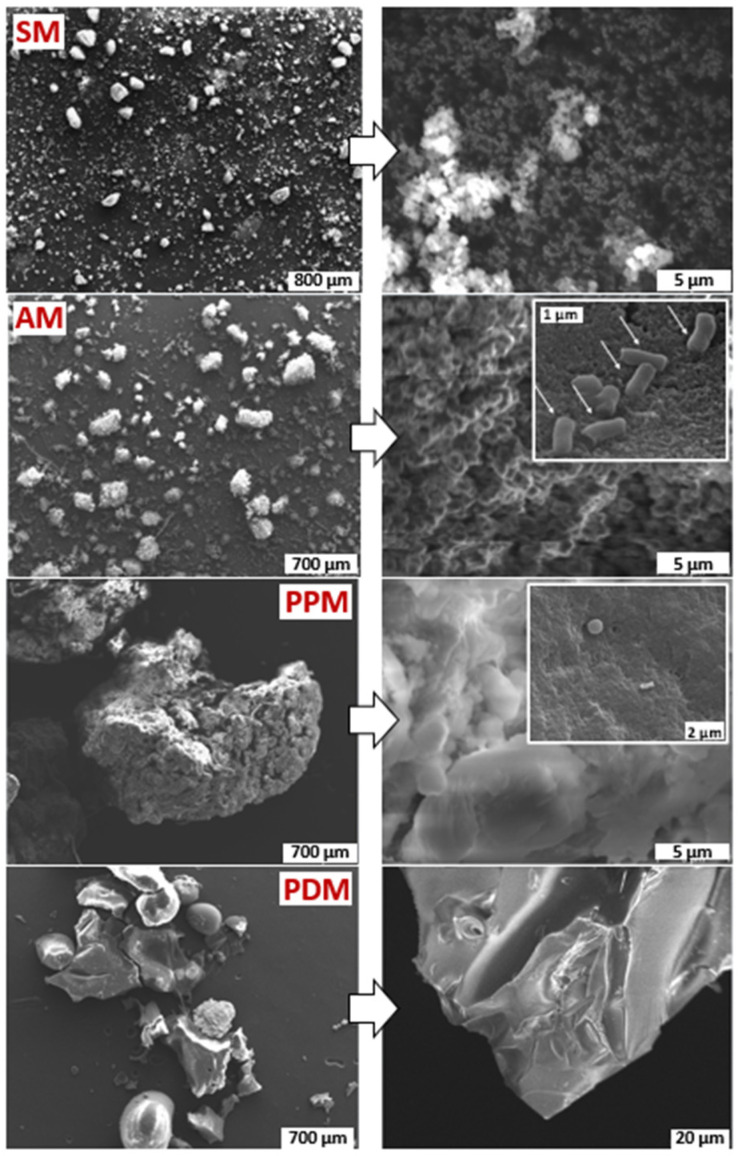
Scanning electron microscopy (SEM) images of SM, AM, PDM, and PPM. AM and PDM were derived and purified from the supernatant of the aqueous media (WSM, cf. Figure 3), while PPM was isolated from cell pellets (PM).

The structural appearance of AM resembled most closely to SM, both showing a fluffy or cloudy-like appearance (Figure 4). Surprisingly, we also found empty cell-like envelops in AM, indicating that (1) the spores of *S. cavourensis* SV 21 must have been freely floating in the growth media resisting centrifugal forces and (2) a melanized layer surrounding the spores conserved its cell-like appearance (see arrows in enlargement of Figure 4) even after intense extraction with several organic solvents followed by a harsh acid precipitation treatment. Our result is in line with previous studies, showing that these black hollow particles preserve morphologies that resemble intact bacterial cells, and thus were termed as “ghost” melanin [25,30]. Compared to AM or SM, PDM and PPM showed a different morphology. While PDM showed a rather uniform amorphous shape, PM offers a conglomerate of different irregular structures that are likely derived from their complex branching and filamentous arrangement as seen in Figure 1D. PDM has a similar morphological structure to insoluble eumelanin isolated from *Bacillus licheniformis* MAL [31].

#### 2.4.3. UV-VIS Absorption Spectra of SM, AM, PDM, and PPM Dissolved in Alkaline Solution

When dissolved in alkaline aqueous solutions (>pH 12), the UV-VIS spectra of AM, PDM, and SM showed similar spectra, absorbing light over the entire tested UV-VIS spectra (230–700 nm). Only PPM showed an enhanced absorption peak at 260 nm (*cf.* Figure 2B) and thus is similar with the UV-VIS profile of the supernatant shown in Figure 2A.

#### 2.4.4. Elemental Analysis of SM, AM, PDM, and PPM

Elemental analysis and chemical characterization of SM, AM, PDM and PPM was performed by two distinct techniques: (1) elemental combustion analysis (EA; Figure 2C, Table S1) that investigates only the relative percentages of carbon (C), nitrogen (N) and sulfur (S) and (2) energy dispersive X-ray analysis (EDX; Figure 2D, Table S2) that is capable to detect all stable elements with the exception of H, He, and Li as well as, in our case, C and N due to oversaturation of the detector. However, by combining both techniques the results shown in Figure 2C,D cover a broad range of elements and depict the similarities and differences between the four samples (for more detail on the elemental composition see Figures S4–S7 and Tables S1 and S2). In brief, the elemental composition was matching closely between SM and AM, while PDM and PPM shared more similarities (*cf*. Figure 2C,D).

SM consisted mainly of C, N (Figure 2C) and to a lesser extend of O, Cl, Fe, S, and Cu (Figure 2D). The finding of S and Cl in black melanin was also reported from sepia melanin [32]. AM consisted also mainly of C and N as well as O, S, Si, Fe, and Cu. However, apart from Cl, the elemental composition from AM closely resembles the one of SM, which also is reflected by a great level of similarities on a morphological level (*cf.* Figure 4). The occurrence of high S percentages in naturally derived melanin was also reported in melanin from *Lachnum* YM404, that was composed of C, H, N, O, and S with content percentages of 60.1, 2.8, 6.5, 14.8, and 14.8, respectively [33]. The presence of Si which we found in AM, has to the best of our knowledge, not been reported before.

Besides C, N, and O, PDM also contained Mg, S, P, Ca, Fe, and Cu (Figure 2D). The elemental composition of PPM resembles the one of PDM but instead of Mg^2+^, Ca^2+^ was dominating the cation composition (Figure 2D). The relatively high proportion of Mg, Ca, Fe, and Cu, which we detected in PDM and PPM, have been reported from sepia melanin [34]. However, PDM and PPM were obtained without the harsh acid-treatment, thus the melanin can be considered as largely intact. Although the free cations and anions were gently removed from the media during purification (i.e., Figure 3), PDM and PPM showed high abundance of divalent cations, where the water-soluble melanin PDM was largely interacting with Mg^2+^ cations and the insoluble form of melanin (PPM) was binding with Ca^2+^ cations. Interestingly, the latter two divalent cations have shown to be interchangeable at the same binding site of melanin [34]. To our knowledge, up to this point there is no study on the effect of Ca or Mg on melanin’s solubility properties. Nonetheless, a study on dissolved organic carbon (DOC) revealed that the addition of divalent Ca ions resulted in a flocculation of up to 50% of the total DOC pool [35]. Thus, we hypothesize that this striking difference in elemental composition, especially the differences of the divalent ions Mg and Ca, may have an important role in the solubility properties of PDM in aqueous solutions.

On the other hand, the loss of most divalent and monovalent ions in SM and AM is likely caused by the harsh acidification process which disrupted the ionic bond and thus releases the Mg or Ca to the media. This rather hard chemical treatment is irreversible and likely changes the physicochemical properties as well as its biological properties tremendously.

#### 2.4.5. Comparing Structural Similarities of SM, AM, PDM, and PPM using RAMAN Spectroscopy

Although the precise molecular structure of melanin is still unknown, RAMAN spectroscopy was used in the past for analyzing melanin in vivo [36,37]. By using low incident laser power (i.e., 458, 515, 633, and 785 nm), rapid spectral acquisition identified two characteristic RAMAN bands at approx. 1380 cm^−1^ and 1580 cm^−1^ that served as a spectral signature for eumelanin [36,38]. A similar signature has also been observed for SM as well as AM (Figure 2E; Figures S8 and S9). SM showed a distinct signal at 488 nm with two broad peaks at around 1380 cm^−1^ and 1590 cm^−1^ and is in line with the results of RAMAN measurement for synthetic derived melanin M8631 (Sigma–Aldrich, Merck, Darmstadt, Germany) reported by [36]. Measurement of SM at higher wavelengths (i.e., 633 and 785 nm) resulted in a slight peak shift or even complete loss of characteristic peaks due to sample burning (*cf.* Figures S8 and S10). Since we achieved best results at 488 nm with SM, RAMAN spectra comparisons of AM, PDM, and PPM were conducted at that wavelength (Figure 2E).

SM showed one distinct RAMAN peak at 1570 cm^−1^ and a broad shoulder between 1300 cm^−1^ and 1450 cm^−1^ (at 488 nm). AM also showed the distinct peak at 1570 cm^−1^, but in addition a clear peak at 1340 cm^−1^. These results confirm that melanin derived from the aqueous media of *S. cavourensis* SV 21 resembles closely with the RAMAN spectra of eumelanin. Similarly, RAMAN spectra of PDM showed a broad peak and in case of PPM a multiple peak pattern that also covered the spectral signature peaks of eumelanin [36,38]. Additionally, PDM and PPM showed two broad peaks at around 950 cm^−1^ and 1120 cm^−1^ (less pronounced in PDM), that were missing in the acid treated melanin samples AM and SM (*cf.* Figure 2E, Figures S11 and S12).

In summary, this comparison highlights that: (1) AM and SM seem to be closely related and thus shows that water-soluble melanin from *S. cavourensis* (both, AM and PDM) shares many structural properties of eumelanin; and (2) PDM and PPM represent likely a more natural source of the bacterial derived melanin since the harsh chemical treatment under low pH likely rearranges the original structure of the pigment. To the best of our knowledge, the RAMAN spectra of PDM and PPM represent an unprecedented characterization of bacterial-derived melanin.

### 2.5. Bioactivities of the Different Melanin Samples

#### 2.5.1. Antioxidizing Capacity, Radical Scavenging Activity of SM, AM, PDM, and PPM, and the Role of Melanin in UV Protection

**Figure 5:**
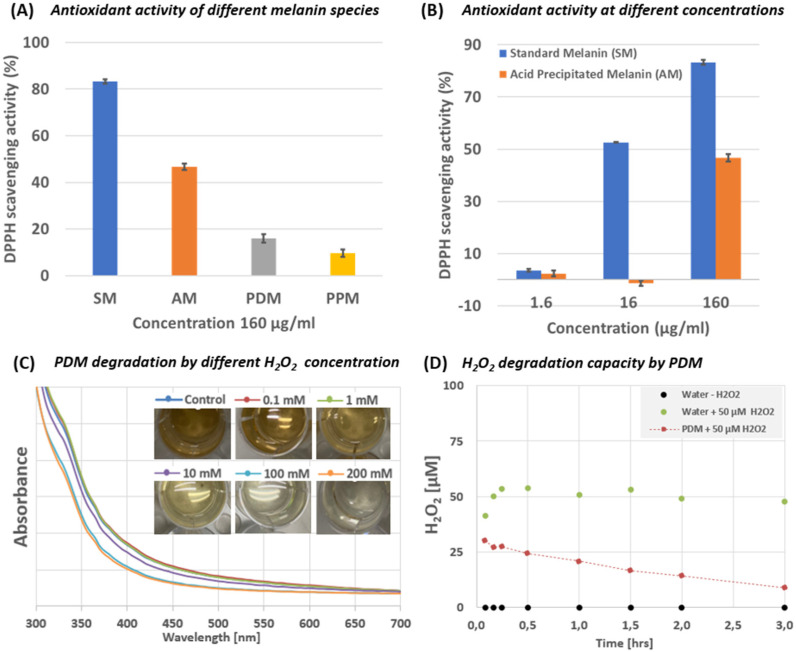
Antioxidant capacity of different melanin types. In (**A**) the DPPH radical scavenging activity of SM, AM, PDM and PPM were tested at a concentration of 160 µg mL^−1^. In (**B**) the DPPH scavenging activities were compared between AM and SM at three different concentrations (i.e., 1.6, 16, 160 µg mL^−1^). In (**C**,**D**) the oxidation potential of PDM was shown using hydrogen peroxide (H_2_O_2_) as oxidation reagent. In (**C**), 5 mL of the dark-brownish aqueous phase of PDM was spiked with different amounts of H_2_O_2_ (final conc.: 0, 0.1, 1, 10, 100, and 200 mM). The absorption spectra of melanin show that H_2_O_2_ bleached the pigment in a concentration dependent manner. In (**D**), the decrease of H_2_O_2_ concentration (50 mM) in the presence and absence of PDM is shown over time.

To test the radical scavenging and antioxidizing properties of the here isolated melanin types we applied two distinct assays. First, we applied the DPPH (2,2-diphenyl-1-picrylhydrazyl) for possible anti-oxidizing, radical scavenging effects in all melanin samples (i.e., SM, AM, PDM, and PPM). Figure 5A shows that acid treated forms of melanin, SM, and AM, showed much stronger radical scavenging ability than the intact melanin derivatives PDM and PPM. Furthermore, this effect is depending on the melanin concentration (i.e., Figure 5B), which confirms previous studies on DPPH scavenging activity of bacterial derived melanin [6,9,10,39,40,41]. Secondly, we spiked PDM with different concentrations of the reactive oxygen species (ROS) hydrogen peroxide (H_2_O_2_; 0, 0.1, 1, 10, 100 and 200 mM H_2_O_2_) while observing (1) the color change from dark brown to colorless as well as monitoring (2) the absorption spectra (300–700 nm) during each experiment (Figure 5C). In a second attempt, we spiked the PDM fraction with 50 µM H_2_O_2_ and observed the decrease of H_2_O_2_ over time (Figure 5D). Both experiments showed a rapid oxidation and bleaching of the melanin pigment and thus its capacity to scavenge H_2_O_2_ out of the environment—a known characteristic of melanin (i.e., [8,42]).

In summary, H_2_O_2_ (Figure 5C,D) and free-radical scavenging activity (DPPH assay; Figure 5A,B) of the different melanin polymers is likely due to the vast abundance of double bonds that are capable to interact with any form of ROS and inhibit possible lethal chain reactions. We propose that the superior antioxidant capacity of melanin has an important role in protecting its producing bacteria such as *S. cavourensis* SV 21 from oxidative substances in the environment.

The production of melanin in an organism (including humans) is mostly related with protection against UV irradiation [43,44]. For example, microorganisms that adapted to extreme sun and UV exposure (i.e., in the Antarctic or high mountains) have also shown to produce elevated levels of melanin [28,45]. Here, we show that melanin also has a protective role against UV irradiation, since the living *S. cavourensis* SV 21 bacteria were able to survive intense UV-C germicidal/bactericidal irradiation (wavelength: 253.7 nm) for the entire tested exposure period of 60 min (Figure S13). The latter result confirms the previously reported protective role of melanin against UV exposure [46,47]. However, we believe that the remarkable survival ability of *S. cavourensis* SV 21 against bactericidal UV irradiation may stem from a combination of melanin coating all producing cells of *S. cavourensis* SV 21, but also the white color of the bacterial surface (cf. Figure 1A,C and Figure S13) which likely reflects most of the incoming irradiation.

Melanin producing bacteria, such as *Vibrio* spp., *Providencia* sp., *Bacillus* sp., *Shewanella* sp., *Staphylococcus* sp., *Planococcus* sp., *Salinococcus* sp., and *Glutamicibacter* sp. have also been isolated from dark pigmented shallow water sponges (e.g., *Haliclona pigmentifera*, *Sigmadocia pumila*, *Fasciospongia cavernosa*, *Spongia officinalis*, and *Callyspongia diffusa*, [48]). Since the production of melanin increased at higher temperatures, melanin may also be involved in the host defense against pathogenic bacteria that have virulence at higher temperatures such as *Vibrio* spp. [49,50]. In addition, some studies have also found melanin in deep-sea marine bacteria. For example, *Pseudoalteromonas* sp. (SM9913), isolated from deep-sea sediments, also produced pyomelanin at a later growth stage and elevated growth temperatures [51].

#### 2.5.2. Potential Antibacterial Role of Melanin Derived from *S. cavourensis* SV 21

**Figure 6:**
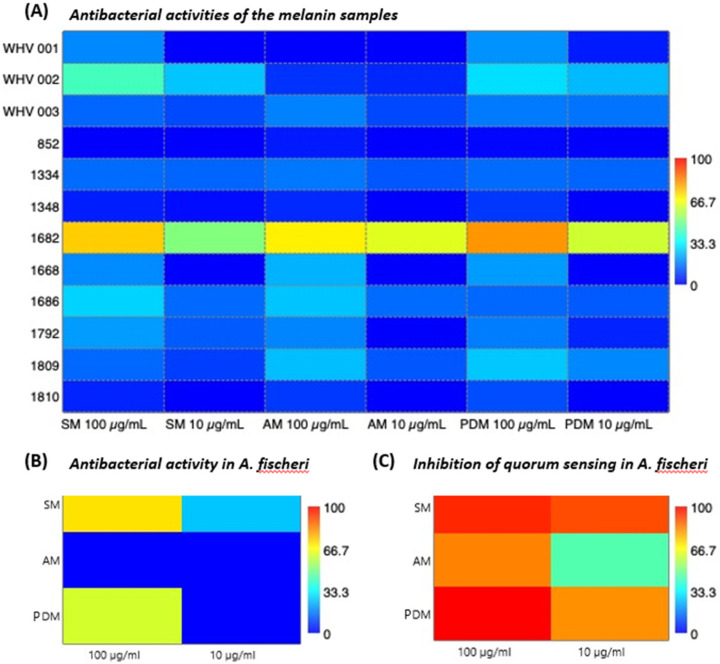
In (**A**) the heat map displays antibacterial activities of the different melanin types SM, AM, and PDM (*x*-axis) towards a test panel of selected environmental bacteria (*y*-axis). The heat map color represents percent antibacterial activity compared to the positive control (10 µM chloramphenicol). In (**B**,**C**) the heat map shows the different melanin types (*y*-axis) tested at two concentrations for their (**B**) antibacterial activity in % and (**C**) inhibition of quorum sensing activity in *A. fischeri*. Positive control for (**B**) 25 µM chloramphenicol and for (**C**) 10 µM furanone. In (**A**) tested bacteria: WHV 001: *Aurantimonas coralicida*; WHV 002: *Vibrio mediterranei*; WHV 003: *Vibrio coralliilyticus*; 852: *Acinetobacter solii*.; 1334: *Aliagarivorans marinus*; 1348: *Vibrio maritimus*; 1682: *Rhodococcus corynebacterioides*; 1668: *Ruegeria areniliticus*; 1686: *Exiguobacterium profundum*; 1792: *Pseudovibrio denitrificans*; 1809: *Ruegeria areniliticus*; 1810: *Pantoea eucrina*.

We also compared antibacterial activities of AM and PDM from the supernatant of *S. cavourensis* SV 21 with SM (Figure 6A). Of the total 12 tested strains only the Gram-positive strain no. 1682 (*Rhodococcus corynebacterioides*) showed growth inhibition to all tested melanin types (i.e., AM, PDM, and SM) in a concentration-dependent manner. PDM showed the highest activity which may be due to its high solubility in aqueous environments. SM also showed minor inhibition against the environmental pathogen no. WHV 002 (*Vibrio mediterranei*), which is known to cause coral bleaching. Although only selectively hindering bacterial growth, our results corroborate previous studies that showed antibacterial activity of soluble and insoluble bacterial melanin against various Gram-positive and -negative bacteria, including *Staphylococcus aureus*, *Bacillus subtilis*, *Enterococcus faecalis*, *Mycobacterium smegmatis*, *Eschericia coli*, *Vibrio parahaemolitics*, and *Pseudomonas aeruginosa* [6,41].

#### 2.5.3. Anti-Quorum Sensing Activity of SM, AM, and PDM

We also screened for activity of SM, AM, and PDM against *Alivibrio fischeri*. The result showed that SM and PDM at concentrations of 100 µg mL^−1^ inhibited the growth of *A. fischeri*, while AM showed no inhibition activity (Figure 6B). At a 10-fold lower concentration of 10 µg mL^−1^, only SM showed weak inhibition and both, AM and PDM did not inhibit the growth of *A. fischeri* at all. However, quorum sensing activity of *A. fischeri* was interrupted more clearly by PDM and SM. To our knowledge, this is the first report on the activity of melanin as a quorum sensing inhibitor. An anti-quorum sensing activity may have ecological importance as a protection strategy by melanin producing strains via interference with the communication ability of competing bacteria. We propose that melanin from *S. cavourensis* SV 21 may have a beneficial ecological function for the microbe–host and/or microbe–microbe interaction. Our results indicated that especially the water-soluble form of melanin PDM was potentially involved in the organism’s defense mechanism by interfering with the quorum sensing capabilities of competing bacteria.

## 3. Conclusions

In this study, the Gram-positive bacterium *S. cavourensis* SV 21 produced two forms of melanin-like pigments: (1) a water-soluble and (2) an insoluble, particulate form. A detailed physicochemical analysis revealed that the latter two pigments share similar physiochemical and biological characteristic compared to a commercially available eumelanin standard. Furthermore, this study provides a novel purification protocol that is not using the common harsh acid hydrolysis treatment, but instead a gentler approach allowing the investigation of the intact form of dissolved (PDM) and particulate melanin (PPM). Our data on PDM and PPM provokes the idea of an important molecular mechanism underlying the solubility of melanin. That is, while the insoluble PPM largely used Ca^2+^ ions, its water-soluble form PDM largely binds to Mg^2+^ ions. We propose that the richness of Mg^2+^ ions attached to the surface of PDM may play an important role in its enhanced solubility in aqueous environments. Furthermore, PDM showed potential bioactivities such as antioxidant, antibacterial, and quorum quenching inhibiting activities. Finally, we believe that from a biotechnological perspective the here described soluble form of melanin may be applicable straight away and thus represents a less expensive melanin source for future applications (i.e., as antibiotic or solar-cell research).

## 4. Materials and Methods

### 4.1. Culture Conditions of S. cavourensis SV 21 and the Experimental Design

The Gram-positive bacterium *S. cavourensis* SV 21 was originally isolated from the sea cucumber *Stichopus vastus* [16]. In this study, we only used marine broth in either its liquid form (marine broth, MB, Carl Roth, Karlsruhe, Germany) or as marine agar plates (MA, prepared from MB by adding 9 g L^−1^ agar; agar-agar Bacteriological, Carl Roth, Karlsruhe, Germany). At the beginning of the experiment, the cryogenic preserved cells of *S. cavourensis* were inoculated on MA plates and kept at room temperature (22 °C) in the dark for 72 h. Subsequently, a single colony of the bacterium (see Figure 1C) was transferred into 20 mL of liquid MB media and incubated at 22 °C for 36 h. About 250 µL of the latter seeding culture, with an OD_600_ of 0.1 (Synergy H1, Biotek, Winooski, VT, USA), was introduced into 200 mL of fresh MB media. Detailed observations of the bacterial colonies on MB media and MA plates were performed by light and fluorescence microscopy (Figure 1B; Zeiss, Axio Scope A1, Oberkochen, Germany).

In this study four different incubation methods for *S. cavourensis* SV 21 were chosen: (1) at room temperature (22 °C) and constant darkness, (2) 22 °C and constant light (Osram L 36 W/21, Munich, Germany), (3) 30 °C and constant darkness, and (4) 30 °C and constant light (Osram L 36 W/21, Munich, Germany). Each of the four experiments was performed in triplicate. Subsamples containing 5 mL of media, individual cells, and cell colonies were taken every 24 h over 14 days and stored in the dark at −20 °C.

### 4.2. Purification of Particulate (PM) and Water-Soluble Melanin (WSM)

After thawing the different time points and replicates, both the supernatant and the particulate biomass of *S. cavourensis* SV 21 were separated by centrifugation at 4 °C at 10,000 rpm for 30 min (Sigma 3–16KL, Sigma Laborzentrifugen GmbH, Osterode am Harz, Germany), which yielded the following two fractions: the insoluble particulate melanin (PM) and the water-soluble melanin (WSM; cf. Figure 3). Subsequently, these two melanin-containing fractions were further purified by removing, inorganic and organic impurities.

#### 4.2.1. AM and PDM

In total, we used two distinct methods for the purification of WSM: (1) by acid precipitation and (2) by dialysis tubing. These two procedures share the first purification step of removing mostly organic impurities by a liquid–liquid extraction step with water-immiscible organic solvents such as n-hexane (Hex), ethyl acetate (EtOAc), dichloromethane (DCM), and butanol (BuOH). After this initial purification (Figure 3), the melanin concentration in the extracted aqueous phase was measured at 340 nm (Synergy H1, Biotek, Winooski, VT, USA) as described in [22].

Melanin in the extracted WSM fraction was precipitated out of the aqueous solution following the method of El-Naggar and El-Ewasy [7] with slight modifications to obtain acid-precipitated melanin (AM). In brief, the darkish, extracted aqueous phase was acidified by adding 6 M HCl solution in a dropwise manner. After successfully adjusting the pH down to pH 2 the solution was allowed to stand for 4 h and the resulting precipitate was collected by centrifugation (10,000 rpm for 15 min). Afterwards, the supernatant was further heated to 100 °C for another 4 h to maximize the precipitation of melanin [8]. After a second round of centrifugation, both precipitates were pooled and washed with distilled water until the pH reached a neutral pH of approximately 7. The extracted and precipitated black pigment was finally lyophilized (Christ Alpha 2–4, Martin Christ Gefriertrocknungsanlagen GmbH, Osterode am Harz, Germany) and stored dry at −20 °C for further chemical and biological characterization.

About 200 mL of the extracted WSM was purified using a dialysis tubing (Servapor^®^ 3500 MWCO, SERVA Electrophoresis GmbH, Heidelberg, Germany) to remove salts and other low molecular weight organic and inorganic compounds to obtain PDM. First, extracted WSM was filled in Milli-Q pre-cleaned dialysis tubing, which was closed on both ends using appropriate water-tight clamps. Subsequently, the WSM-filled tubes were dialyzed in a 2 L Milli-Q reservoir with constant exchange of the Milli-Q every 4 h. After 48 h, the conductivity of the Milli-Q reservoir was measured using a multi-parameter portable meter (MultiLine^®^Multi 3630 IDS, Weilheim, Germany) until it reached 1–1.5 µs/cm for the entire 4 h period. Finally, the dialyzed pigment was also lyophilized and the black residual pellets were stored dry at −20 °C for further chemical and biological characterization.

#### 4.2.2. Purified Particulate Melanin (PPM) from PM

After its separation from the supernatant, the pellet was extracted sequentially using a range of non-polar and polar organic solvents (i.e., Hex, EtOAc, DCM, methanol (MeOH), and BuOH). After each extraction step (or washing step) with the respective solvent, the PM was centrifuged at 10,000 rpm for 15 min and the organic phases removed. Furthermore, the extracted PM was fully dried under a stream of nitrogen and washed three times with Milli-Q to remove water-soluble compounds such as inorganic salts. The purified particulate melanin (PPM) was finally lyophilized and stored at −20 °C for further chemical and biological characterization.

### 4.3. Analysis of the Melanin

#### 4.3.1. UV-VIS Profile

The UV-VIS spectra of the individual samples were measured in a flat bottom, UV light permeable 96-well plate (Greiner Bio One International GmbH, Kremsmünster, Austria) using a microplate reader (Synergy H1, Biotek, Winooski, VT, USA). All dried melanin samples (i.e., PM, WSM, AM, PDM, and PPM) were dissolved in a strong basic solution (pH = 12) using a 1 N concentrated NH_4_OH solution. All UV–VIS absorption spectra were compared with a commercially available synthetic melanin standard (SM) that was also dissolved in the same basic solution (M 8631; Sigma-Aldrich, Merck KGaA, Darmstadt, Germany). The spectra of the different samples were recorded in 1 nm steps in the range of 230 nm to 700 nm.

#### 4.3.2. Scanning Electron Microscope (SEM) Coupled to Energy-Dispersive X-Ray (EDX) Analysis

Morphology and structure of the bacterial colonies and the melanin samples were examined by SEM (Hitachi S-3200N, Krefeld, Germany). Furthermore, the elemental compositional pattern of the different melanin samples (in their solid form) were analyzed with EDX-System (Oxford INCA System Penta-FET Precision INCA X-Act, 10 mm^2^ SDD Detector).

#### 4.3.3. Elemental Combustion Analysis

The C, N, and S analysis was carried out by the CHNS-Elemental-Analyzer Vario EL Cube (ELEMENTAR Analysesysteme GmbH, Langensbold, Germany). The instrument was calibrated with sulfanilic acid. Oxygen dosage was set to 10 mg/120 s. Combustion tube temperature was set to 1150 °C. 1 mg of sample was weighed in a tin cap and mixed with 40 mg of tungsten oxide. Finally, all samples and standards were placed in an autosampler. The percentages of C, N, and S as well as all remaining uncharacterized elements (UK for unknown) were compared.

#### 4.3.4. Solubility Properties of the Different Melanin Samples

To calculate the solubility properties of the different dried melanin samples (i.e., PPM, AM, PDM) and the synthetic melanin standard (SM, M8631; Sigma-Aldrich, Merck KGaA, Darmstadt, Germany), 1 mg of each type was transferred separately into Eppendorf tubes which each contained 1 mL of the following organic solvents: *n*-hexane, DCM, *n*-butanol, MeOH. The samples were also tested for solubility in Milli-Q at neutral (pH = 7), acidic (pH = 2), and basic (pH = 12) conditions. Eppendorf tubes were centrifuged for 20 min at 4 °C and 10,000 rpm to observe whether the melanin samples were insoluble, partly soluble, or fully dissolved.

#### 4.3.5. RAMAN Spectroscopy

Dried melanin samples (i.e., PPM, AM, PDM, SM) were placed on a metal holder and measured using three laser energies with the following wavelengths: 488, 633, and 785 nm. Raman measurements were performed with a Bruker Senterra Raman microscope/measurement system (Bruker, Billerica, MA, USA). Many variations of laser powers, integration times, accumulations were tested to achieve results without damaging the samples. Samples shown in Figure 2E (Figures S8–S12) were measured with laser at a 488 nm under the following conditions: 10× magnification, 50 × 1000 µm aperture, and resolution of 3 to 9 cm^−1^ at laser power of approximately 5 mW. PPM and PDM were measured with 1 s integration time and 20 accumulations. AM was measured with 1 s integration time and 40 accumulations. SM was less prone to damage but had a very weak Raman signal, so it was measured with a 100 s integration time with three accumulations. Baseline corrections were performed using the advanced baseline correction tool in the software SpectraGryph 1.2.14 [52]. This software uses an adaptive baseline correction to remove the fluorescent background from the Raman signals. It should be noted that the fluorescent background of samples PPM and PDM samples is about an order or magnitude larger than Raman signal, thus making the Raman signal difficult to extract.

### 4.4. Bioactivities of the Samples

#### 4.4.1. Antioxidant and Radical Scavenging Activity

The PDM fraction was spiked with different concentrations of hydrogen peroxide (H_2_O_2_). The oxidative effect of different H_2_O_2_ concentrations (0–200 mM) on PDM was monitored by measuring the absorption wavelength of melanin at 340 nm [22]. For that, 100 µL of the aqueous solution were transferred to a 96-well plate (Greiner UV-star, flat bottom, Greiner Bio One International GmbH, Kremsmünster, Austria) and analyzed using a microplate reader (Synergy H1, Biotek, Winooski, VT, USA).

To monitor the concentration of H_2_O_2_ over time, the Amplex^®^ UltraRed (Fischer Scientific GmbH, Schwerte, Germany) assay was used. In brief, a H_2_O_2_ stock reagent was prepared by adding 340 µL DMSO to one mg of Amplex^®^ UltraRed. Then, 50 µL of the stock reagent was mixed with 100 μL of 10 U mL^−1^ horseradish peroxidase (HRP) and 4.85 mL of Milli-Q water. 50 µL of the latter reagent mix was added to 50 µL of the aqueous analyte, which was shortly incubated for 2 min at room temperature in the dark. The fluorescence was measured every 30 min at wavelength settings of 530/590 nm (excitation/emission) in the microplate reader. The concentrations of H_2_O_2_ in the melanin containing aqueous solutions as well as in positive (Milli-Q water + 50 µM H_2_O_2_) and negative controls (Milli-Q water) were determined from a H_2_O_2_ standard curve.

Antioxidant activity of the different melanin types was also determined using the free radical 2,2-diphenyl-1-picrylhydrazyl (DPPH) scavenging assay. Samples from SM, AM, PDM, and PPM were prepared to 200 µg mL^−1^ with MeOH. Additionally, SM and AM were diluted to 20 and 2 µg mL^−1^, respectively. 200 µL of sample were added to 50 µL of a 1 mM DPPH solution (in MeOH) in a microplate well, and then mixed vigorously by pipetting multiple times. The mixture was then incubated for 30 min. Each sample was tested in triplicate. Since melanin could not be completely dissolved in the test system and possibly interfered with the absorption, a subsample of 150 µL was taken from each well and transferred into a new well after melanin particles had settled. The absorbance (A) of the sample with DPPH was measured at 520 nm. The scavenging activity was determined by comparing the absorbance of the sample with the absorption of a control sample that contained only MeOH and DPPH.

(1) $$\%\;\mathrm{inhibition}=\frac{\left(A\;\mathrm{control}-A\;\mathrm{sample}\right)}{A\;\mathrm{control}}\times 100\%$$

#### 4.4.2. Antibacterial Activity of the Different Melanin Samples

Antibacterial activity of the melanin samples (i.e., PPM, AM, PDM, SM) were measured by analyzing growth of a microbial test panel consisting of 12 environmental bacteria (Figure 6). For that, the optical density (OD) of the media of each individual bacterium was measured at 600 nm (OD_600_) using a Synergy H1 Microplate Reader (BioTek, Bad Friedrichshall, Germany). Test panel of bacteria were prepared and grown in liquid MB media overnight at 22 °C and continuously shaken at 120 rpm. At T_0_ cultures were diluted until an OD_600_ of 0.01 was reached. Melanin samples were dissolved in 1N NH_4_OH to achieve two solutions with the concentration of 10 and 1 mg mL^−1^. 2 µL of each solution were added and diluted with 198 µL of the bacterial culture to achieve a final concentration of 100 and 10 µg mL^−1^. 2 µL of the 1N NH_4_OH solution added to the 198 µL of the bacterial culture was used as negative control. The positive control was chloramphenicol at a concentration of 10 µM. The potential antibacterial activity of the melanin samples was determined after 5 h of incubation at 22 °C and 120 rpm.

#### 4.4.3. Anti-Quorum Sensing Activity

*Alivibrio fischeri* was used as the model organism to test for quorum sensing (QS) activity in this study. The bacterium was cultured overnight at 22 °C in Hastings medium (HS, Na_2_HPO_4_.12H_2_O 9.35 g, KH_2_PO_4_ 1 g, (NH_4_)_2_SO_4_ 0.5 g, MgSO_4_.7H_2_O 0.21 g, NaCl 30 g, trypton 5 g, yeast extract 3 g, glycerol 2 mL, agar 18 g, aqua dest. ad 1 L, pH 7.4). Initial cell density for the starting point was diluted to OD_600_ of 0.01. Melanin samples were prepared as above. Chloramphenicol (25 µM) and 5-(bromomethylene)-2H(5H)-furanone (10 µM) were used as positive control for antibacterial and QS inhibition activity, respectively. Media and the 1N NH_4_OH were used as blank and negative control, respectively. The luminescence activity was measured in a Synergy H1 Microplate Reader (BioTek, Bad Friedrichshall, Germany) by using a suitable white microplate (BRANDplates^®^ pureGrade™ S, BrandTech Scientific, Inc., Essex, UK). Besides luminescence, cell density was measured at OD_600_ nm until a signal intensity of 10^5^ RLU (relative light unit) was reached in the negative controls. Results from the anti-quorum sensing as well as antibacterial activity were visualized using matrix plots in Past 4.02 (Paleontological Statistics, https://folk.universitetetioslo.no/ohammer/past/) with 100 as a maximum value.

#### 4.4.4. Survival of the Melanin Producing *S. cavourensis* SV 21 Strain after Germicidal UV-C Irradiation

UV-C irradiation was done in a clean bench (EnvairEco^®^ Safe Basic Plus, Envair Ltd., Lancashire, UK) equipped with an UV-C lamp emitting a wavelength of 253.7 nm (Sankyo-Denki, 30 Watt, Kanagawa, Japan). The bacterium was plated onto MA plates that were only covered half with aluminum foil (the covered half was used as control). After irradiation of 15, 30, 45, and 60 min, plates were incubated at 22 °C in an incubator in the dark and colony growth observed every 24 h for 4 days.

## Figures and Tables

**Table 1:** Solubility properties of SM, AM, PDM, and PPM in different organic and inorganic solvents.

	Tested Organic Solvents (Increasing in Polarity from Left to Right)				Tested Inorganic Solutions		
Sample	n-Hexane	DCM	n-Butanol	MeOH	Milli-Q	Acidic Cond. (pH 2)	Alkaline Cond. (pH 12)
**SM**	-	-	-	+	-	-	+++
**AM**	-	-	-	+	-	-	+++
**PDM**	-	-	-	-	+++	+++	+++
**PPM**	-	-	-	-	+	+	+

Abbreviation: Acid-based melanin (AM); purified particulate melanin (PPM); purified dissolved melanin (PDM); synthetic melanin standard (SM); dichloromethane (DCM); and methanol (MeOH). Annotations: (-): insoluble; (+): partly soluble; (+++): fully soluble.

## Data Availability

The EDX and RAMAN spectra data are available in the Supplementary Materials.

## Supplementary Materials

The following supporting information can be downloaded at: https://www.mdpi.com/article/10.3390/md20010054/s1, Figure S1. Building blocks of melanin from the tyrosine pathway; Figure S2. (A) and (B) show seven sequential supernatants (day 1, 3, 5, 7, 9, 11, and 13) of a 14-day incubation period of *S. cavourensis* SV 21; Figure S3. pH of the culture of *S. cavourensis* SV 21; Figure S4. EDX spectra of SM; Figure S5. EDX spectra of AM; Figure S6. EDX spectra of PDM; Figure S7. EDX spectra of PPM; Figure S8. Raman spectra of SM in three different wavelengths: 488 nm (top), 633 nm (middle), and 785 nm (bottom); Figure S9. Raman spectra of AM at three different wavelengths: 488 nm (top), 633 nm (middle), and 785 nm (bottom); Figure S10. RAMAN spectra of SM at three wavelengths: 488, 633, and 785 nm; Figure S11. Raman spectra of PDM at three different wavelengths: 488 (top), 633 (middle), and 785 nm (bottom); Figure S12. Raman spectra of PPM at three different wavelengths: 488 (top), 633 (middle), and 785 nm (bottom); Figure S13. The *Streptomyces cavourensis *strain SV21 incubated for 4 days after irradiated with UV-C (254 nm) for: (A) 5 min, (B) 30 min, (C) 60 min; Table S1. Elements in AM, DM, PM, and SM; Table S2. C, N, S, and UK (unknown) percentage from elemental combustion analysis.
