# Supplementary material for: Characterization of an Insoluble and Soluble Form of Melanin Produced by Streptomyces cavourensis SV 21, a Sea Cucumber Associated Bacterium
Source: Mar Drugs. 2022 Jan 6;20(1):54. doi: 10.3390/md20010054 (PMC8777673; doi:10.3390/md20010054)
Supplement: Supplementary file 1 [file marinedrugs-20-00054-s001.zip › marinedrugs-1466669-supplementary.pdf]

Supplementary Information

Figure S1. Building blocks of melanin from the tyrosine pathway.

Figure S2. (A) and (B) show seven sequential supernatants (day 1, 3, 5, 7, 9, 11, and 13) of a 14-day incubation period of *S. cavourensis* SV 21 after centrifugation and after extraction with organic solvents (*cf.* Figure 3). This experiment includes four different experiments at (**A**) 22 °C and (**B**) 30 °C either incubated under constant light stress (A_1 and B_1) or in complete darkness (A_2 and B_2). In (**C**) the light absorbance recorded at 340 nm was measured in the supernatant for all seven timepoints and experiments.

Figure S3. pH of the culture of *Streptomyces cavourensis* SV 21.

Figure S4. EDX spectrum of SM.

Figure S5. EDX spectrum of AM.

Figure S6. EDX spectra of PDM.

Figure S7. EDX spectra of PPM.

Figure S8. Raman spectra of SM in three different wavelengths: 488 nm (top), 633 nm (middle), and 785 nm (bottom).

Figure S9. Raman spectra of AM in three different wavelengths: 488 nm (top), 633 nm (middle), and 785 nm (bottom).

Figure S10. RAMAN spectra of SM at three wavelengths: 488, 633, and 785 nm.

Figure S11. Raman spectra of PDM in three different wavelengths: 488 nm (top), 633 nm (middle), and 785 nm (bottom).

Figure S12. Raman spectra of PPM in three different wavelengths: 488 nm (top), 633 nm (middle), and 785 nm (bottom).

Figure S13. The *Streptomyces cavourensis* strain SV21 incubated for 4 days after irradiated with UV-C (254 nm) for: (A) 5 minutes; (B) 30 minutes; (C) 60 minutes.

Table S1. C, N, S, and UK (unknown) percentage from elemental combustion analysis (EA).

Table S2. Presence and absence of Elements in SM, AM, PDM, and PPM.

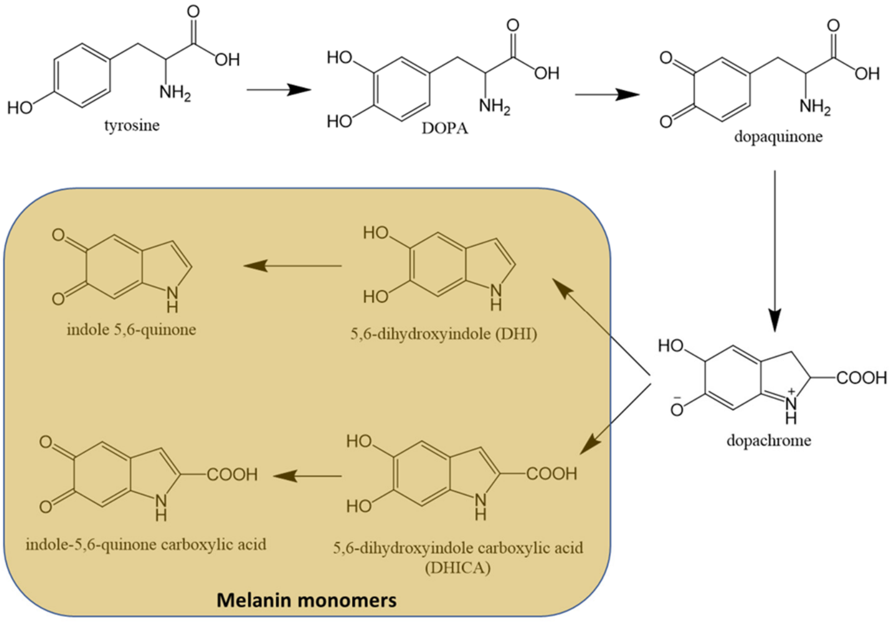

Figure S1. Building blocks of melanin from the tyrosine pathway.

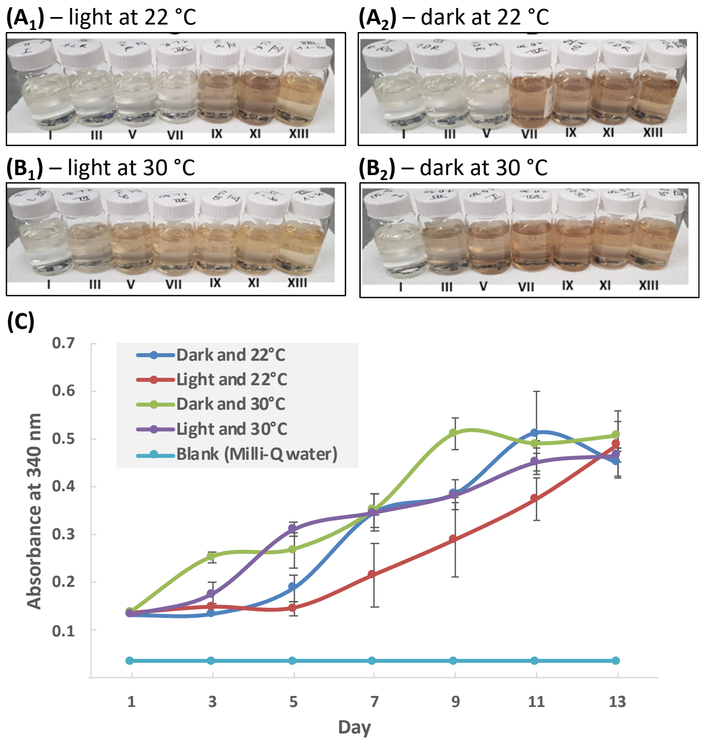

Figure S2. (**A**) and (**B**) show seven sequential supernatants (day 1, 3, 5, 7, 9, 11, and 13) of a 14-day incubation period of *S. cavourensis* SV 21 after centrifugation and after extraction with organic solvents (*cf.* Figure 3). This experiment includes four different experiments at (**A**) 22 °C and (**B**) 30 °C either incubated under constant light stress ($\mathbf{A}_1$ and $\mathbf{B}_1$) or in complete darkness ($\mathbf{A}_2$ and $\mathbf{B}_2$). In (**C**) the light absorbance recorded at 340 nm was measured in the supernatant for all seven timepoints and experiments.

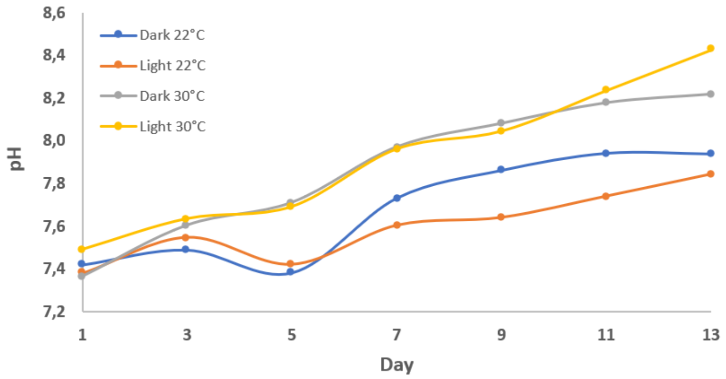

Figure S3. pH of the culture of *Streptomyces cavourensis* SV 21.

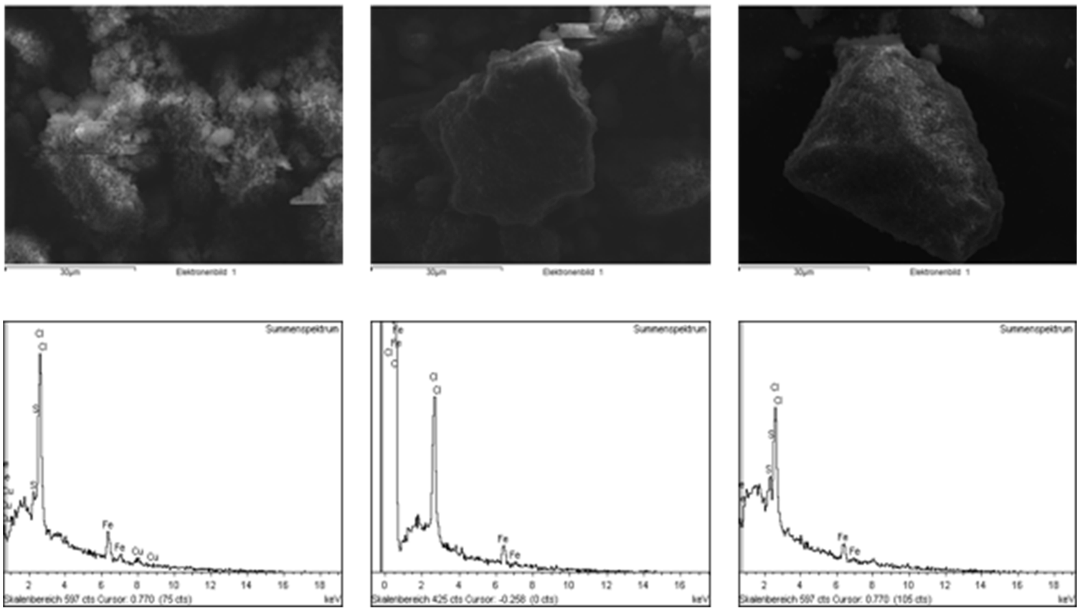

Figure S4. EDX spectrum of SM.

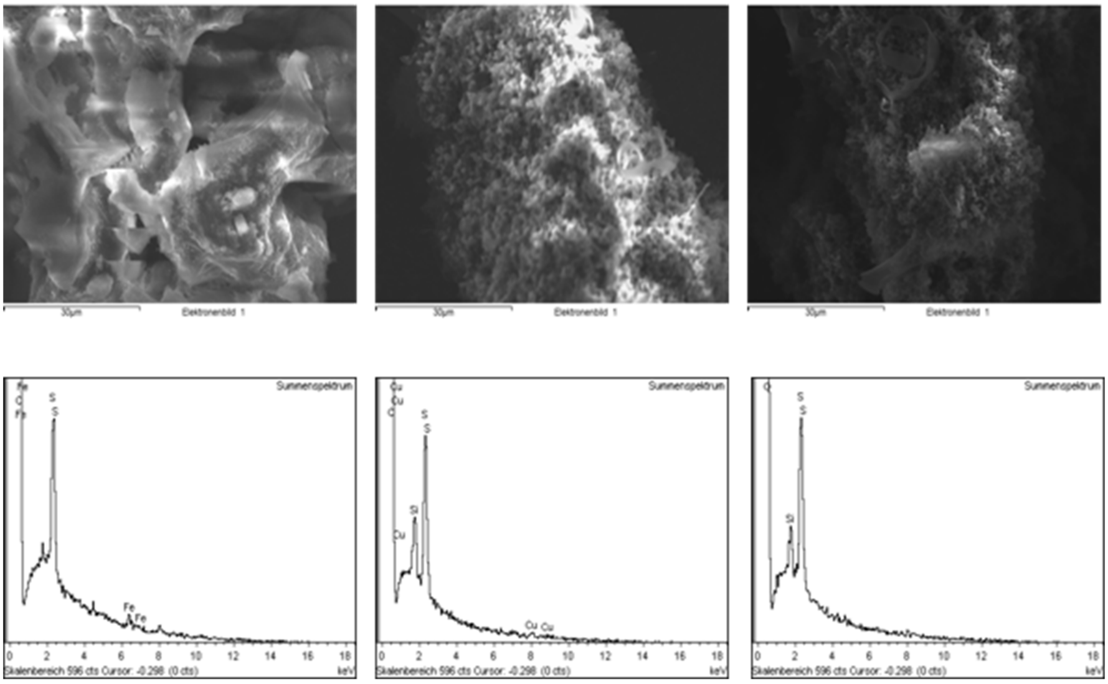

Figure S5. EDX spectrum of AM.

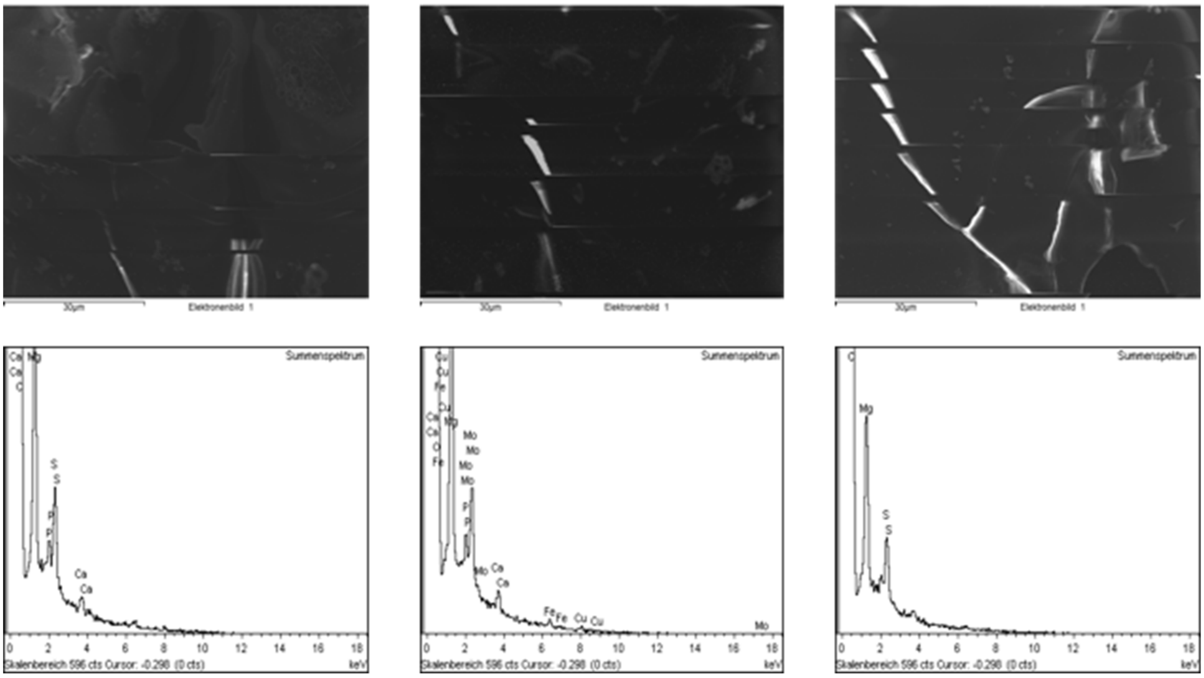

Figure S6. EDX spectra of PDM.

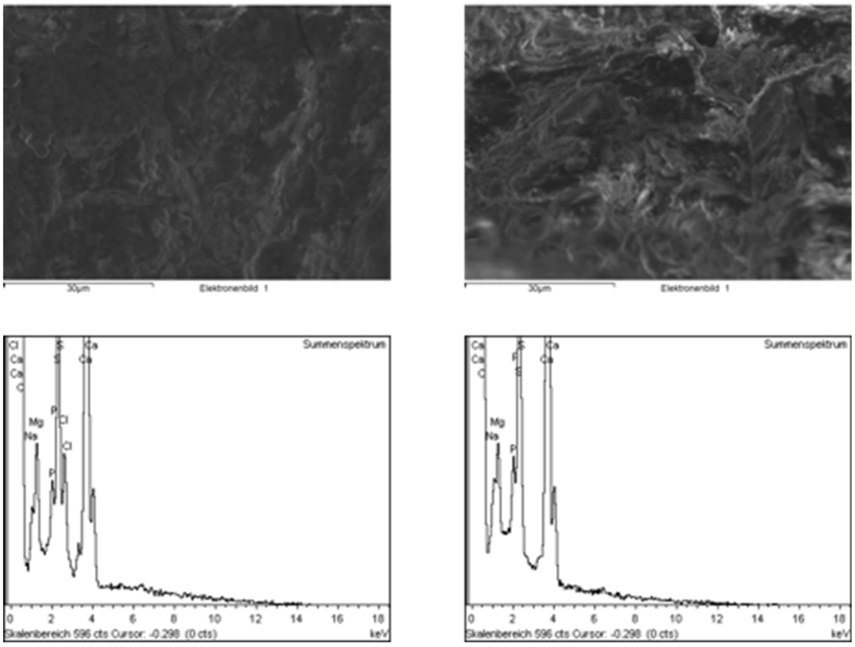

Figure S7. EDX spectra of PPM.

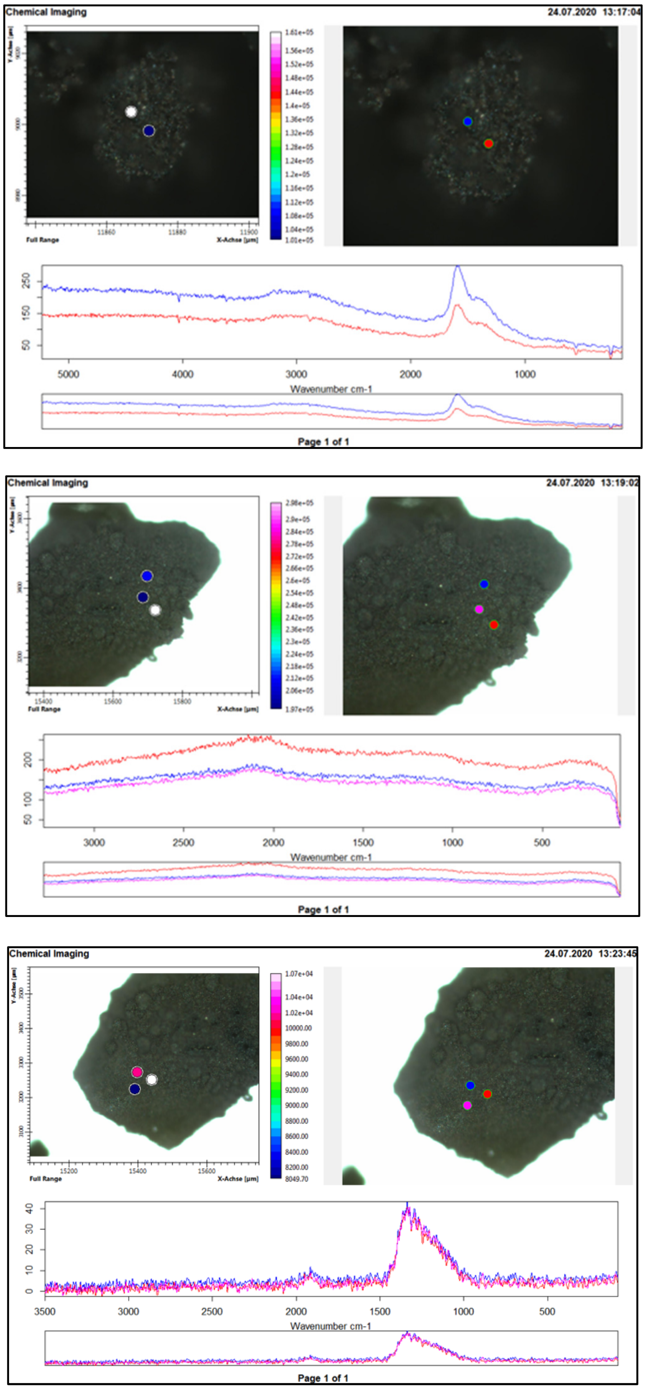

Figure S8. Raman spectra of SM in three different wavelengths: 488 nm (top), 633 nm (middle), and 785 nm (bottom).

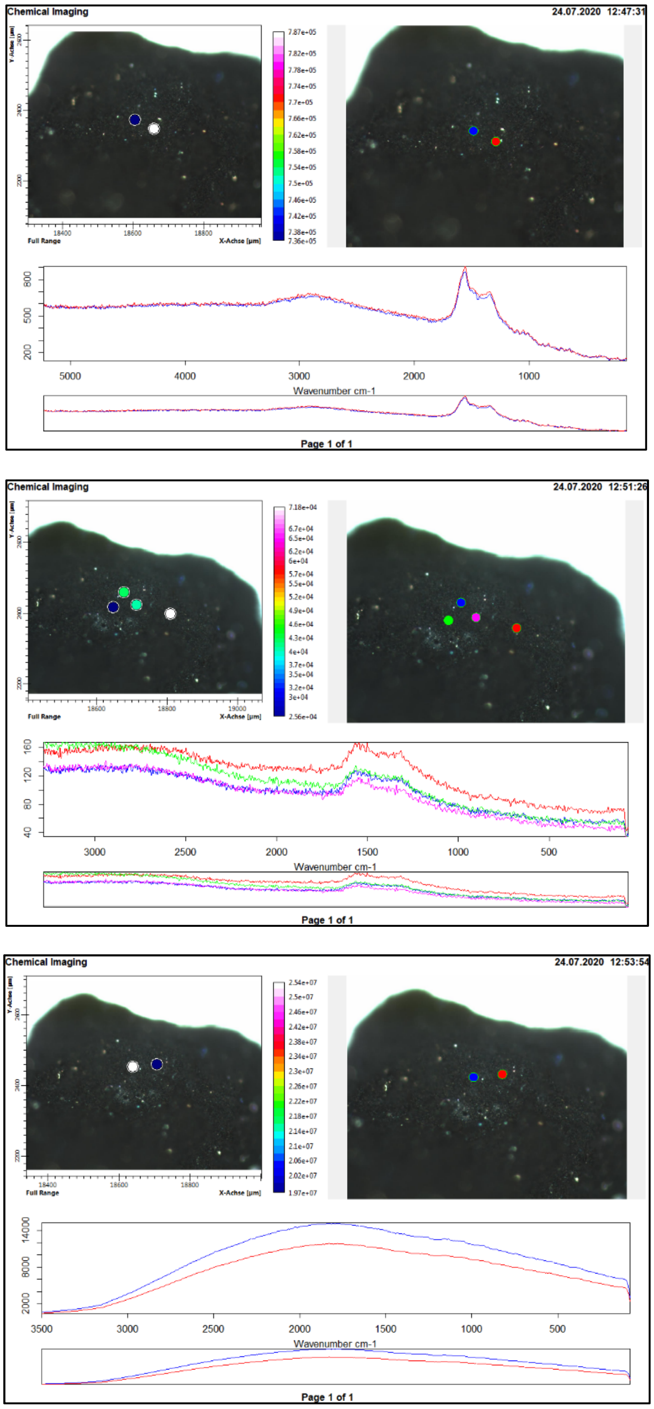

Figure S9. Raman spectra of AM in three different wavelengths: 488 nm (top), 633 nm (middle), and 785 nm (bottom).

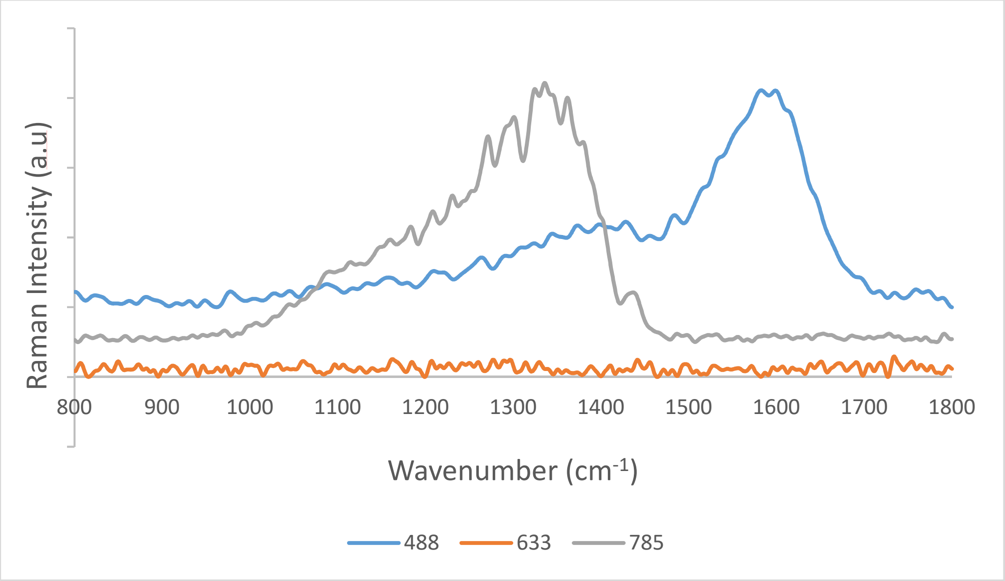

Figure S10. RAMAN spectra of SM at three wavelengths: 488, 633, and 785 nm.

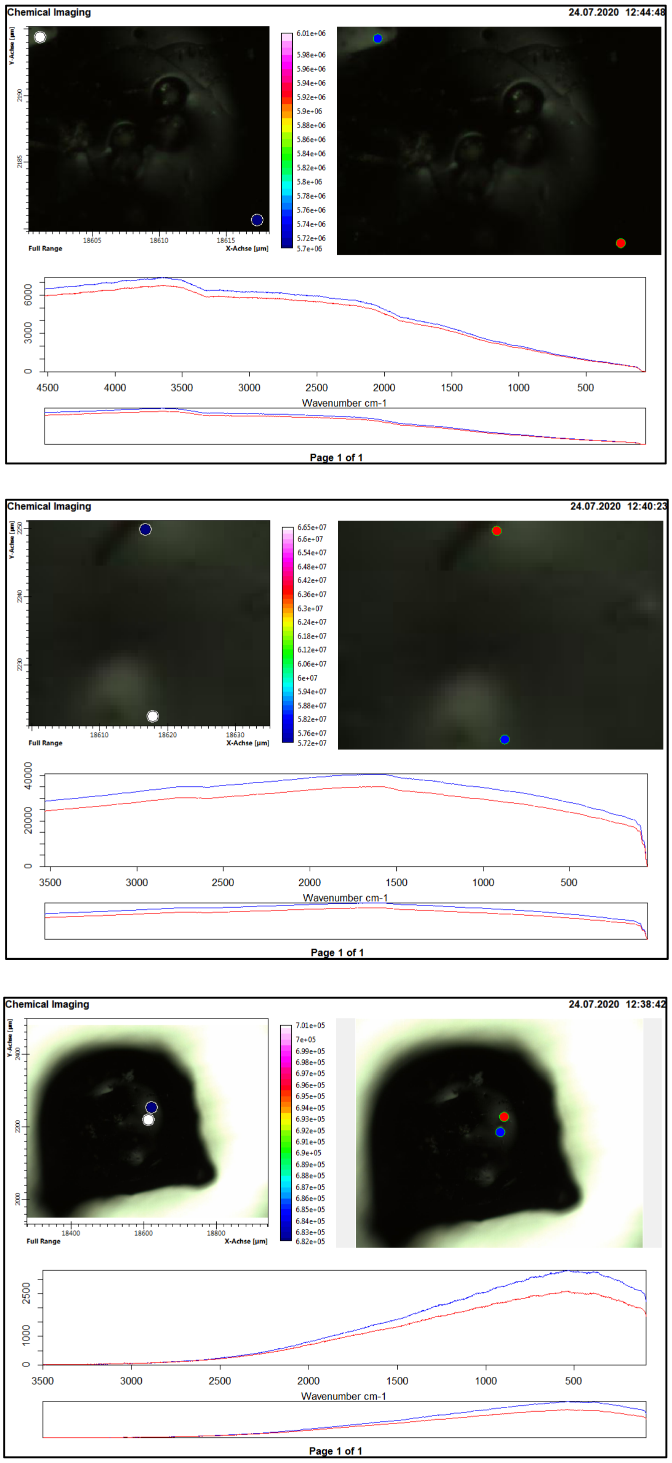

Figure S11. Raman spectra of PDM in three different wavelengths: 488 nm (top), 633 nm (middle), and 785 nm (bottom).

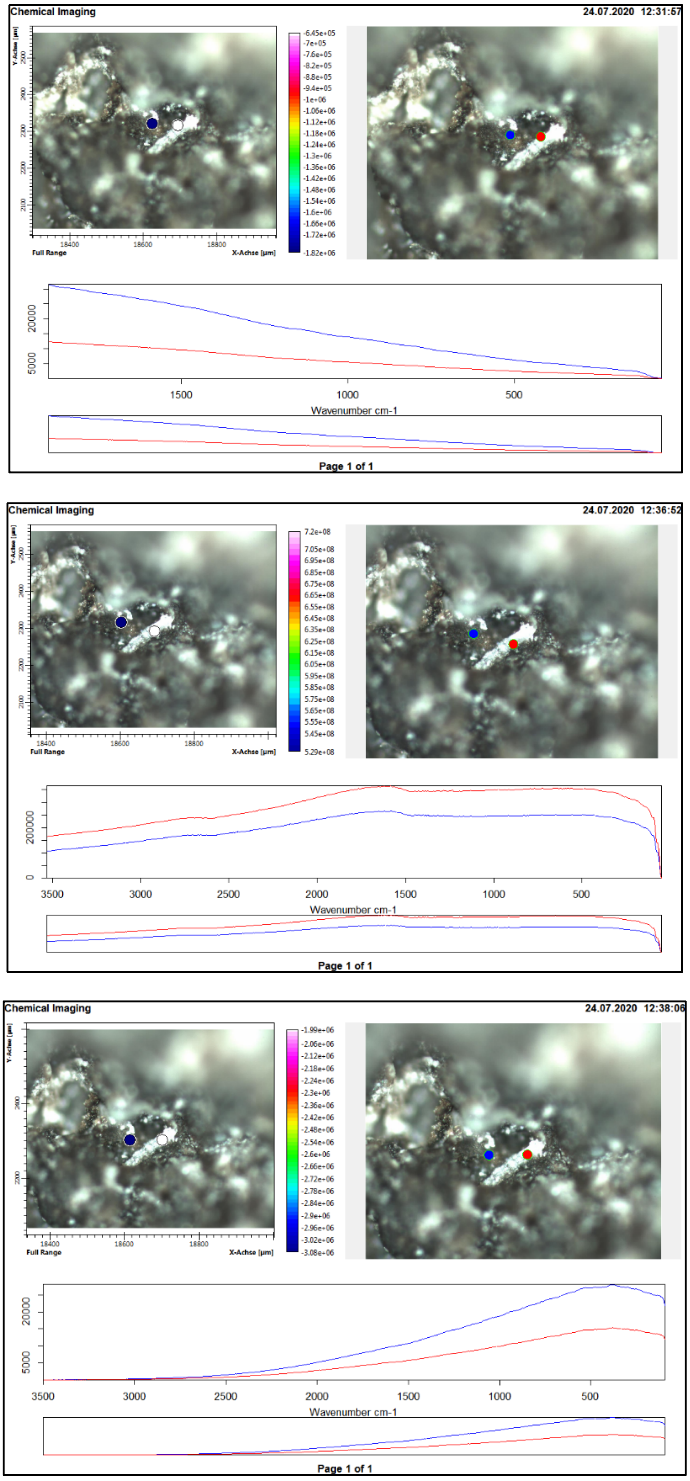

Figure S12. Raman spectra of PPM in three different wavelengths: 488 nm (top), 633 nm (middle), and 785 nm (bottom).

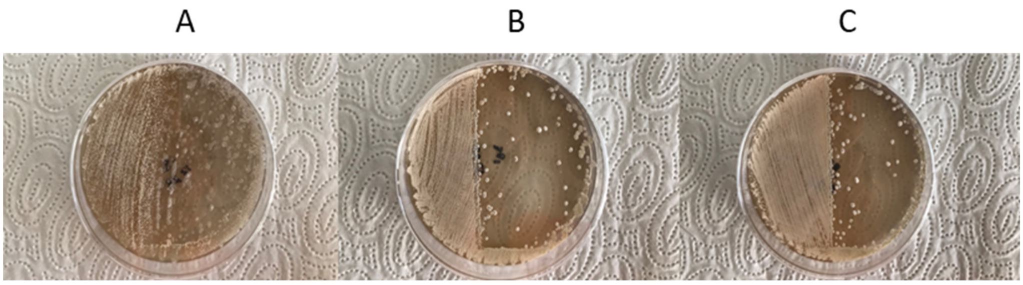

Figure S13. The *Streptomyces cavourensis* strain SV21 incubated for 4 days after irradiated with UV-C (254 nm) for: (A) 5 minutes; (B) 30 minutes; (C) 60 minutes.

Table S1. C, N, S, and UK (unknown) percentage from elemental combustion analysis (EA)

No.	Sample	N [%]	C [%]	S [%]	Unknown
1.	SM	6.27	50.08	0.74	42.91
2.	AM	12.75	52.15	1.55	33.55
3.	PDM	9.82	37.92	1.23	51.03
4.	PPM	4.69	24.74	2.99	67.58

Table S2. Presence and absence of Elements in SM, AM, PDM, and PPM

Elements	SM	AM	PDM	PPM
Na	-	-	-	√
Mg	-	-	√	√
Si	-	√	-	-
S	√	√	√	√
P	-	-	√	√
Cl	√	-	-	√
Ca	-	-	√	√
Fe	√	√	√	-
Cu	√	√	√	-
